# Can visiting the site of death be beneficial for bereaved families after terror? A qualitative study of parents’ and siblings’ experiences of visiting Utøya Island after the 2011 Norway terror attack

**DOI:** 10.1080/20008198.2018.1463795

**Published:** 2018-04-25

**Authors:** Pål Kristensen, Kari Dyregrov, Atle Dyregrov

**Affiliations:** a Center for Crisis Psychology, Faculty of Psychology, University of Bergen, Bergen, Norway; b Master’s Program in Mental Health and Substance Abuse Care, Western Norway University of Applied Sciences, Bergen, Norway

**Keywords:** Terrorism, visiting death site, bereavement, family, grief, complicated grief, prolonged grief, terrorismo, visitar el lugar de la muerte, duelo, familia, aflicción, duelo complicado, duelo prolongado, 恐怖主义, 访问死亡地点, 丧亲, 家人, 悲伤, 复杂的悲伤, 延长哀伤, • Visiting the site of death was associated with several benefits such as facilitating acceptance of the reality of the loss.• Distressing reactions, such as intrusive thoughts and re-enactment images, were common, especially at the actual place of death.• Having the opportunity for multiple visits appeared to optimize the benefits.

## Abstract

**Background.** After the 2011 terror attack on Utøya Island, a collective visit was organized for bereaved families. There is limited knowledge whether bereaved families can benefit from such visits after terror.

**Objective.** This study aims to explore how bereaved families experienced visiting the site of death after the 2011 terror attack.

**Method.** As part of in-depth interviews, 22 parents and 16 siblings were asked whether they had visited Utøya and, if so, how they experienced the visit. Participants’ responses were analysed using thematic analysis.

**Results.** The results showed that for the majority of the bereaved, visiting Utøya had been important in processing their loss. Three key themes emerged as to what they considered important with the visit: ‘seeing the actual place of death’, ‘seeking factual information’, and ‘learning to know the island’. These factors were associated both with beneficial reactions (e.g. accepting the reality of the loss increased cognitive clarity) and with distressing reactions (e.g. intrusive thoughts, re-enactment images), but the benefits had outweighed the distress. Having the opportunity for multiple visits seemed to optimize the benefits.

**Conclusion.** Bereaved families should be offered the opportunity to visit the site of death after terror.

## Abstract (es)

**Planteamiento.** Después del ataque terrorista de 2011 en la isla de Utøya, se organizó una visita colectiva para las familias en duelo. Existe un conocimiento limitado de si las familias en duelo pueden beneficiarse de tales visitas después del hecho aterrador.

**Objetivo.** Este estudio tiene como objetivo explorar cómo las familias en duelo experimentaron la visita el sitio de la muerte después del ataque terrorista de 2011.

**Método.** Como parte de las entrevistas en profundidad, se les preguntó a 22 padres y 16 hermanos si habían visitado Utøya, y en caso afirmativo, cómo experimentaron la visita. Las respuestas de los participantes se analizaron mediante análisis temáticos.

**Resultados.** Para la mayoría de los familiares en duelo, visitar Utøya había sido importante para procesar su pérdida. Aparecieron tres temas clave sobre lo que consideraban importante de la visita; ‘Ver el lugar real de la muerte’, ‘buscar información objetiva’ y ‘aprender a conocer la isla’. Estos factores se asociaron tanto con reacciones beneficiosas (por ejemplo, aceptar la realidad de la pérdida), como con reacciones dolorosas o angustiantes (p. ej.,pensamientos intrusivos, imágenes de reescenificación). Tener la oportunidad de realizar varias visitas pareció optimizar los beneficios.

**Conclusión.** Las familias en duelo deberían tener la oportunidad de visitar el lugar de la muerte después de un hecho aterrador.

## Abstract (zh)

**背景：** 2011年在Utøya 岛发生恐怖袭击事件后，遇难者家属组织了一次集体访问。还不清楚丧失亲人的家庭是否可以从恐怖之后的这种探视中获益。

**目的：**这项研究旨在探讨死难者家属在2011年恐怖袭击事件后如何经历死亡现场。

**方法：**深入访谈的一部分，有22位家长和16位兄弟姐妹被问及是否访问了Utøya，如果是的话，他们的访问经历如何。使用专题分析分析参与者的反应。

**结果：**对于大部分丧亲者，拜访Utøya对于处理他们的丧失感非常重要。他们认为重要的三个关键主题是：‘看到实际的死亡地点’，‘寻求事实信息’，以及‘学习了解这个岛’。这些因素与有益反应（例如，接受损失的现实），以及痛苦反应（例如，侵入性想法，重演影像）相关联。有多次访问的机会似乎优化了这些好处。

**结论：**应该让丧失亲人的家庭有机会在恐怖事件之后探访死亡地点。

## 1. Introduction

It is well documented that losing a loved one in a terror incident can have significant and long-lasting mental health consequences for the next-of-kin (Norris & Wind, 2009). High prevalence of prolonged grief disorder (also termed complicated grief), posttraumatic stress disorder (PTSD), mood disorders, and reduced functioning in daily life have been reported across different incidents and populations (Dyregrov, Dyregrov, & Kristensen, 2014; Neria et al., 2007, 2008; Pfeffer, Altemus, Heo, & Jiang, 2009; Pfefferbaum et al., (2001); Shear, Jackson, Essock, Donahue, & Felton, 2006). It is therefore necessary to acquire more and better knowledge of how we can help bereaved families cope with their loss.

In the early phase after a violent loss, it is important for the bereaved to grasp the reality of the death and to accept that the death was inevitable given the circumstances (Kristensen, Weisæth, & Heir, 2012). This can be facilitated by getting information about the circumstances of the death (Winje, 1998) and participating in different grief rituals such as viewing the deceased and attending the funeral (Chapple & Ziebland, 2010; Singh & Raphael, 1981). Grief rituals can make the loss more real and can give the bereaved an opportunity to say a final goodbye (Reid & Reid, 2001). Studies have shown that rituals can decrease anxiety and alleviate grief by helping the bereaved regain a feeling of control (Brooks et al., 2016; Norton & Gino, 2014).

Recent studies after large-scale accidents and natural disasters have shown that bereaved families can benefit from visiting the place where the death of a loved one occurred (Kristensen & Franco, 2011; Kristensen, Tønnessen, Weisæth, & Heir, 2012). A study after the 2004 Southeast- Asian tsunami showed, for example, that seeing the disaster area made it easier to realize why it was impossible for their loved one to survive, which had reduced troublesome ruminations (Kristensen et al., 2012). Many also felt closer to their loved one at the site. Some studies suggest that revisiting the site after trauma can reduce posttraumatic stress symptoms (Heir & Weisæth, 2006; Murray, Merritt, & Grey, 2015a). Grief rituals and visits to the site of death can be complementary but different ways to confront the reality of traumatic deaths. While rituals are a symbolic interpretation of the actual experience, making the death less harsh through the use of symbols and metaphors (Romanoff & Terenzio, 1998), visiting the death scene and walking in the precise footsteps of the deceased’s final moments is a more realistic and actual or direct experience of the death, which may also be more painful.

Our knowledge of how bereaved families experience visits to the site of death after terror is scarce. The aim of this study is to explore how bereaved family members’ experience visiting Utøya after the 2011 terror killings in Norway. Based on earlier studies (Kristensen & Franco, 2011; Kristensen et al., 2012), we aim to explore if a visit can be beneficial in the grieving process and/or a distressing experience.

## 2. Organization of collective visits to Utøya island for bereaved families after the terror killings in 2011

When a major national or international disaster strikes, national authorities may decide to organize a collective visit for bereaved families to the site of death. In other cases, the company or organization responsible for the safety of those who were killed may arrange a visit for bereaved families, or families have gathered at such sites themselves (Eyre, 2007; Kristensen et al., 2017). After the terror attack in 2011 at Utøya Island in Norway, where a single perpetrator killed 69 young adults and adolescents, and around 500 survived, a national expert group recommended that bereaved families be offered the opportunity to visit the island (Report IS-1984E, 2012). It was deemed important that bereaved families be given the opportunity to visit the island before others, particularly the media. The first collective visit was organized one month after the attack, and a second visit a month and a half later for those who were not able to participate the first time. The aim of these visits was to show the bereaved families the site where their loved one was found killed, to receive brief information from police, and perform private rituals at the site. Prior to the visits, there was much consideration and debate about how to present the different sites, as the killings were spread throughout the island. Some (e.g. the police) wanted to shield the bereaved from traces of the attack and recommended hiding/repairing the bullet holes in the walls. In the end, it was decided not to cover the bullet holes, to retain the colouring from the blood seeping into the wooden floors, as well as to leave other damages caused by the perpetrator. This was recommended by mental health professionals in order for bereaved family members to see the sites in their actual condition to the extent possible after the killings and thereby reduce the sense of disbelief.

Personal effects that were not damaged were removed, washed, and presented to the bereaved in a respectful manner with great care. The bereaved could visit a building at a nearby location where all identifiable objects were placed on 69 tables covered by white cloths. In a separate room, unidentifiable effects were kept so that the bereaved could search for the personal items of the deceased. All floors were washed prior to the arrival of the families.

Written information was sent out to the bereaved families in advance specifying what they would see at the site and informing them about common reactions both during and after the visit. Photographs of the buildings and rooms where the killings occurred were displayed on the two embarking locations used to transport the bereaved to the island, in order to optimize the mental preparation for what they would encounter at the island. Several health teams were mobilized and present at the island to assist those who needed physical/mental assistance during the visit.

On the first collective visit, approximately 360 next-of-kin were present, representing 60 of the 69 deceased. Each family was escorted by two police officers and a volunteer from the Red Cross to ‘their’ site. However, since many of the deceased were killed at the same place (e.g. 14 adolescents were killed near ‘Pumpehuset’), it took many hours for all the bereaved families to visit their site. At the site, the police said where the deceased were found and answered any question that the family had related to the death. Flowers were available for everyone, and the buildings had sandboxes where the families could place candles. Later, it was possible to visit the island at anniversaries and on open days. Individual visits have also been allowed.

## 3. Aims and research focus

The aims of this paper are as follows:

1. Explore how the bereaved families experienced visiting the site of death after the 2011 terror attack on Utøya Island. Is it beneficial and/or distressing?
2. Identify factors that made the visit beneficial or distressing.

## 4. Method

### 4.1. The project

This study is part of the project, ‘Bereaved parents, siblings and friends after Utøya, 22 July 2011ʹ, a longitudinal mixed-method study initiated following the terror attack in Norway, 22 July 2011 (Dyregrov et al., 2014). The study was conducted at the Center for Crisis Psychology in Bergen, Norway, and data was collected 18 (T1), 28 (T2), and 40 months (T3) after the terror incident. The data presented in this paper were collected at T2 and consist of in-depth interviews with parents and siblings.

The project was approved by the Regional Committees for Medical and Health Research Ethics in Norway.

### 4.2. Procedure and participants

The names of parents and siblings of the deceased were accessed through the National Population Register. The interview sample was drawn from a total sample of parents (*N* = 57) and siblings (*N* = 36) who consented to be interviewed. Since the aim was to explore the diversity of experiences, we selected the sample according to different background variables (i.e. gender, age, place of residency, variations in symptoms, education, any additional children, age of the deceased). The sample selected for interviews consisted of 22 parents (11 mothers, 11 fathers) and 16 siblings (11 sisters, 5 brothers). The age of the parents was between 40 and 61 years (Mean = 50.1), whereas the age of the siblings was between 15 and 44 years (Mean = 24.3). Geographically, both the parents and the siblings represented all parts of Norway. The parents were fairly well educated; 64% had a higher education (beyond 12 years). The age of the deceased ranged between 15–21 years (*M* = 17.5).

### 4.3. In-depth interviews

A theme guide consisting of four main themes was constructed for the interviews: 1. Psychosocial impact of losing a child or sibling, 2. Circumstances influencing the loss, 3. Help and support, and 4. Self-help strategies. Under the third theme (Help and support), we asked the following questions: Have you been to Utøya after the terror attack? If so, can you describe your experience of the visit(s). Depending on the answers, we explored conditions associated with experiencing the visit(s) as beneficial and/or distressing.

All parents and siblings were interviewed by the first and second author in their homes or at places chosen by the interviewees between October and December 2013. To synchronize the interview method, the second author conducted a trial interview in the presence of the first author. This interview was discussed prior to conducting other in-depth interviews. The length of the interviews varied between two and five hours, including required or desired breaks. The interviews were audiotaped and fully transcribed.

None of the interviewees had been to Utøya before the terror attack, and 20 of 22 parents (91%) and all 16 siblings (100%) had visited Utøya after the terror attack. The majority of parents (80%) and siblings (75%) had visited the island more than once. Most had participated in collective visits and anniversaries the first and second year after the attack, but many had also visited the island on their own (parents: 30%, siblings: 73%).

### 4.4. Analyses

Parents’ and siblings’ descriptions of how they experienced visiting Utøya were analysed according to thematic analysis (Braun & Clark, 2006). All meaningful units related to visits to Utøya were identified in the interviews, which were read and re-read several times. Statements with similar content were marked, coded, and named before they were categorized into main and subthemes. The qualitative analyses were conducted by the first and second authors, and then adapted in line with consensus discussions between all authors.

## 5. Results

Almost all participants had visited Utøya Island and underlined that the visit had been an important part of processing the loss. Three key themes were highlighted from their narratives of what they considered important with the visit: ‘seeing the actual place of death’, ‘seeking factual information’, and ‘learning to know the island’. These three key themes were associated with both beneficial and distressing reactions (subthemes).

## 6. Seeing the actual place of death

The first key theme, ‘seeing the actual place of death’, was related to a need to see what the site looked like. Many struggled with difficult fantasies of the site before the visit, or had strong feelings of disbelief related to the loss.

### 6.1. Accepting the reality of the death/correcting disturbing fantasies

Seeing the place of death was, for many, important in order to grasp or realize that the death had occurred. A sibling said:

The first visit was very important. I had a huge need to visit Utøya to try to grasp or comprehend that he in fact had died, that his heart stopped there.

During the first visits to the island, the bereaved could see concrete traces of the killings, e.g. bullet holes, blood stains on the floor inside the buildings. Witnessing these traces was painful, but also important in trying to grasp what had happened and to counteract the strong sense of disbelief. One sibling said:

It was important for me to see where she had been killed, it was important for me to be where she was the last few seconds of her life. So it helped a little on understanding what happened. It made it a bit more concrete, a little more real when I saw the bullet holes, saw the place she was found. But it was also terribly painful.

Many mentioned that they needed to visit Utøya several times to really comprehend what had happened, but the traumatic circumstances of the death made the process difficult. A sibling said:

And I was there before the trial, it early spring, and I had to see the island, when it was not summer, without media attention, and to spend more time there. And I felt that it was part of comprehending what had happened, of working through the loss. I felt it was important. Still, I was always reminded when I was there of how he died. So it was painful to be there, but it was also good to be there.

Others struggled with difficult fantasies of what the site looked like, and experienced that the visit had changed these disturbing images. A sibling said: ‘I had many pictures in my head of what happened that day, and they were not always correct (…) so when I visited Utøya these pictures changed’.

### 6.2. Feeling closer to the deceased

Another beneficial effect of seeing the place of death was feeling closer to the deceased. Several bereaved experienced a special connection to the place of death at the site. One sibling said: ‘Both the island and the place where he died becomes very special to me, and I immediately felt attached to it’. Some actively sought closeness at the site, for example by touching the ground where the deceased was found, or by being on the place of death on the exact time when their loved one was killed. For some the site became sacred, and they said that they wanted to have the opportunity to visit the place of death also in the years to come. Subsequently, they felt much anger and resentment towards the owner of the island when they, shortly after the killings, recommended that several of the buildings should be destroyed, a decision that was later reversed.

### 6.3. Establishing a common family narrative

Several parents said that visiting the island together with family members had been important when talking about the incident. The joint family visit had imparted a common family narrative or understanding of Utøya and what had occurred there. One mother said that it was difficult to explain to other family members what it felt like to visit Utøya. She noted the following:

On some of the visits, we brought along other family members so that they also could see the island and understand more how we feel when we are there. This has been important for us, but also for them. Now we have something in common, and they can better relate to what we are saying when we talk about Utøya.

For some siblings the joint family visit had some challenges. This was mainly due to the responsibility and concern they felt for their parents, since they had not been able to focus on themselves during the visit. One sibling put it like this: ‘It was quite difficult because I had to relate to my parents’ grief when I had enough with my own’.

### 6.4. Activation of trauma and grief reactions

Being at the site not only gave the bereaved a feeling of closeness to their loved one, they also came closer to the gruesome acts of violence that happened there. Many experienced intrusive thoughts or images of the killings at the site. A sibling said: ‘It was difficult, particularly to see the site where he was murdered. I could imagine more clearly what had happened to him when he was killed’. Others said that they could almost feel the deceased’s suffering and fear while being at the site. A sibling said:

It is very distressing to be on the actual site, that’s where she lived her last minutes, and that’s where she experienced the most horrible things you can ever imagine. It hurt to realize and feel that.

These distressing reactions were, for some, also related to becoming more aware of the magnitude of the incident, realizing the number of all who were killed at the island, and what they went through. A sibling said:

What became clearer to me at the visit was all the other things that happened on the island – the other adolescents who were killed, all the screams, all those who hid, all those who were afraid – I now have more images of these in my mind.

For others, the visit activated grief reactions such sadness and yearning. This was particularly difficult for those who actively had tried to avoid their grief as a way to cope with the loss. A sibling said: ‘It is hard for me to visit the site because I cannot control my feelings anymore when I am there. Then I am confronted by my own grief, and that’s what I am struggling with’. Some also blamed themselves when they saw the site. A parent said that he had talked to his son on the phone only minutes before he was killed. He said:

I told my son that he should hide, but he said that there were no places to hide (…) When I visited Utøya, I saw that he was right; there were no places to hide there. And I realized that I had given him the worst advice I could give.

Although most of these reactions were temporarily activated while being at the site, a few experienced a worsening of reactions after they had visited Utøya. A parent said that her nightmares had changed for the worse after seeing the place of the death. She said:

After I had seen the place where the death of my daughter occurred, my nightmares became more concrete. I had seen the landscape and the exact place, and then it got worse because they became more physical. Now I saw and recognized the place of the killings in my nightmares.

## 7. Seeking factual information

The second key theme, ‘seeking factual information’, was also, for many, a reason to visit Utøya. Most of the bereaved had many unanswered questions related to what had happened and had a need for information.

### 7.1. Filling in the blanks

At the first collective visit the police informed each family on the site what they knew about each child/sibling. This was painful, but also helpful. A mother said:

The first visit was the most difficult one. Then we were told his (the deceased’s) story, where he ran, and where he was found killed – all the information about what had happened. But at the same time, it also felt good. Now we had seen the place, instead of ruminating about what may have happened there.

For some, visiting Utøya together with a person who had been on the island on the day of the killings had given access to detailed information of where their loved one had been, and the difficult choices that they had to take. In this sense the ‘survivor’ could fill in some of the blanks and unanswered questions that many bereaved struggled with. A mother said:

It was very important to meet with Kristin, who had been together with our daughter just minutes before she was killed. Then I managed to understand why they had stayed so long at the site. First, I thought that hiding there was like being trapped, but after I talked to Kristin I realized that it was impossible for me to understand what it was like being in that situation.

## 8. Learning to know the island

The third key theme, ‘learning to know the island’, was related to getting a more concrete impression of the whole island and to try to comprehend the choices their loved one had made, which many had ruminated about.

### 8.1. A different view of the island

In their effort to understand and put more ‘pieces in the puzzle’, many bereaved stated that they had to walk all over the island. This had given a different view of what it looked like. One father said:

When we looked at photographs of the island, we got a quite different view of the topography and distances than what it actually was. When we were there, we realized, for example how small the island was.

A mother said: ‘The first time I visited Utøya, I did not really understand anything’. Having the opportunity to walk around the island had changed her perceptions of it. She said further:

Prior to the visit I had all these ideas of how it looked like at Utøya, the different rooms, where they could hide, distances and so on. So when I walked around the island I got a different picture. I understood more why my son did what he did, and how difficult it was to hide.

### 8.2. Following the escape route facilitated an understanding

Furthermore, in trying to get answers to their questions, many followed the ‘route’ where their loved one had escaped to see with their own eyes where they had tried to hide from the perpetrator. This ‘reconstruction’ had given a better understanding of the choices that they had to make. A mother said:

I stood on the path, and looked down towards where my son had hidden. Then I realized that he had found a good hiding place. I had ruminated a lot about this, and I couldn’t have seen this on a picture (...) After the second visit to Utøya, things fell more into place, and I felt calmer from within. I am sure it made it easier for me to go on.

### 8.3. Integrating the deceased’s perspective of the island

Visiting the island had facilitated ‘integration of the deceased’s perspective’ of the island. Several parents expressed that they more easily could realize why their child wanted to go to the camp, and why they thought it was so much fun to be there. One father said: ‘The second time we were there, Utøya was more like how we imagined the adolescents experienced it, with summer and nice weather, and you could realize how fantastic a time they must have had there’. Another father said: ‘I think that the positive experience we had at the island the second time we were there pushed the bad things away. Then I got another impression of what they actually enjoyed when they were there’.

## 9. Discussion

In this paper, we explored how bereaved families experienced visiting the site of death after a terror incident. The main finding from the in-depth interviews is that visiting the site of death is beneficial but is also associated with painful and distressing reactions. Still, the benefits had outweighed the distress. The need to know what the site look like and to see in order to comprehend or make sense of what has happened was for many the major motive for visiting the site (Gilles & Neimeyer, 2006). Achieving a precise perception of the physical surroundings of the site of death seems to give a more concrete and detailed understanding and realization of the death or what we call increased cognitive clarity (Dyregrov, Straume, & Saari, 2009). For some this had corrected misinterpretations and reduced troublesome ruminations. These findings are consistent with other disaster studies (Kristensen & Franco, 2011; Kristensen et al., 2012), and suggest that these may be universal experiences independent of the type of disaster.

The findings can inform both grief and trauma theory. Generally the bereaved are drawn towards places that are linked with the deceased (Parkes, 1970) and, according to attachment theory, searching for the deceased is a common initial behavioural response to try to recover their loved one. William Worden’s task model underlines that the first task is to accept the reality of the loss, and posits that grief rituals can facilitate such acceptance (Worden, 2006). As we have seen from this study, seeing the place of death can have a powerful effect in counteracting disbelief and facilitate acceptance of the reality of the loss, especially after traumatic deaths.

Cognitive trauma theory posits that a central component of processing a traumatic event is facilitating integration of the trauma into autobiographical memory (Ehlers & Clark, 2000). Subsequently, trying to build a comprehensible overall picture from fragmented pieces of the whole (filling in the gaps) is considered essential for processing such losses. In this sense, visiting the site can provide a more coherent understanding and narrative of the event instead of striving with fragmented images about what has happened (Heir & Weisæth, 2006).

In addition to cognitive processing of the loss, our findings also show that visiting the site of death can strengthen relational bonds in the family. Making sense of the loss always happens within the context of the family (Nadeau, 2008). As we have seen, family visits seem to facilitate adaptation to a traumatic loss through a shared acknowledgement of the reality of the loss, clarifying facts about the death, and through a shared experience of the loss (Walsh, 2007).

The finding that seeking out the last place where a person lived can give an increased feeling of closeness to the deceased is consistent with the theory of continuing bonds (Klass, Silverman, & Nickman, 1996). However, maintaining a continuing and positive bond at the site of death can be difficult, especially when being there activates thoughts and images about the gruesome acts of violence. This is the essence of traumatic grief, when positive reminiscing is disturbed by the way the death occurred (Barlé, Wortman, & Latack, 2017). That many experienced intrusive thoughts and re-enactment images related to the deceased’s suffering at the site (Rynearson, 2018) suggests that the need to know about what happened to their loved one may have a downside. This raises the question whether this kind of confrontation is necessary or even advisable for all bereaved in order to cope with a violent loss, but also that it is a delicate balance between what is therapeutic and what is traumatic. For some, visiting the site of death may primarily be a painful reminder of the loss. Still, we lack systematic knowledge of who suffer the most during a visit, and if it even can be harmful for some.

## 10. Limitations

This study has some limitations. We do not know the representativeness of those who consented to be interviewed. Although persons bereaved by single-incident violent losses underline their need for visiting the death scene (Williams, Rheingold, McNallan, & Knowlton, 2018), more research is needed to see whether our findings can be repeated after violent losses such as from suicide, accidents, or homicides. The coding and consensus discussions during the analyses were conducted by three researchers with extensive experience in the bereavement field. However, it is possible that other researchers may have categorized the material differently.

## 11. Implications

Our findings suggest that having the opportunity for multiple visits can optimize the benefits. Survivors who were directly exposed to the disaster can contribute with important information about the deceased’s last moments, but this can also be an extra burden on the survivors. Subsequently, more research is needed before this can be used systematically in the follow-up of bereaved families. Furthermore, careful planning, mental preparation prior to the visit, and support through the visit is necessary to try to minimize or reduce some of the distress that can be associated with such visits (Kristensen et al., 2017; Murray, Merritt, & Grey, 2015b). Regardless of preparation, we believe that some reactivation of reactions must be expected.

Visiting the site of death may be a useful intervention for those who struggle with prolonged grief and PTSD (Murray et al., 2015a). Cognitive-behavioural theories of grief suggest that maladaptive cognitions, such as ruminations about the circumstances of the death and phobic avoidance of loss reminders, is associated with the development or maintenance of prolonged or complicated grief (Boelen & Smid, 2017). A visit to the site of death can be considered an in-vivo exposure to the reality of the death, and can reduce negative or maladaptive ruminations (Eisma & Stroebe, 2017).

There are still research questions related to visits to the site of death that need to be addressed such as the number of visits to recommend, why some refuse to visit the site of death, and if there is anyone who should refrain from visiting.

## 12. Conclusion

Bereaved families should be offered the opportunity to visit the site of death after terror, but tailored preparations are necessary to reduce some of the distress that is associated with such visits. Having the opportunity for multiple visits can optimize the benefits.
